# Global prevalence of metabolic syndrome among patients with type I diabetes mellitus: a systematic review and meta-analysis

**DOI:** 10.1186/s13098-021-00641-8

**Published:** 2021-03-02

**Authors:** Rebuma Belete, Zerihun Ataro, Ahmedmenewer Abdu, Merga Sheleme

**Affiliations:** 1 Department of Medical Laboratory Sciences, College of Health and Medical Sciences, Haramaya University, Harar, Ethiopia; 2 School of Medicine, College of Health and Medical Sciences, Haramaya University, Harar, Ethiopia

**Keywords:** Prevalence, Metabolic syndrome, Type 1 diabetes mellitus, Systematic review, Meta-analysis

## Abstract

**Background:**

The presence of metabolic syndrome among diabetes patients is frequent and is associated with an increased incidence of chronic complications and mortality. Despite several studies have been conducted, there is no overall estimation on the prevalence of metabolic syndrome among type 1 diabetic patients. Therefore, this study aimed to estimate the pooled prevalence of metabolic syndrome among patients with type 1 diabetes mellitus.

**Methods:**

Medline via PubMed, CINAHL, ScienceDirect, Ovid, Google Scholar, ResearchGate and African Journals Online were searched by limiting publication period from January 2005 to October 2020. Data were extracted with a standardized format prepared in Microsoft Excel and exported to Stata 16.0 for analyses. The I^2^ statistic was used to check heterogeneity across the included studies. DerSimonian and Laird random-effects model was applied to estimate pooled prevalence and 95% confidence interval across studies. Funnel plot symmetry, Begg’s test and Egger’s regression test were used to determine the presence of publication bias. Subgroup and sensitivity analysis as well as meta-regression were conducted to explore the potential sources of heterogeneity. The study protocol is registered on PROSPERO with reference number: CRD42020213435.

**Results:**

In this meta-analysis, a total of 27 studies with 45,811 study participants were included. The pooled prevalence of metabolic syndrome was 23.7% with substantial heterogeneity (I^2^ = 98.2%; P < 0.001). Geographical-based subgroup analysis revealed that the highest prevalence was observed in Australia (27.3%). As per meta-analysis of 17 studies, the pooled prevalence of metabolic syndrome in female type 1 diabetes patients (25.9%) was slightly higher than male T1DM patients (22.5%).

**Conclusion:**

Nearly a quarter of the type 1 diabetes mellitus patients were affected by metabolic syndrome. Therefore, more attention should be paid to the prevention and control of the epidemic and for the reduction of the morbidity and mortality associated with metabolic syndrome among type 1 diabetes mellitus patients.

## Background

The metabolic syndrome (MetS) also called Syndrome X and Insulin Resistance Syndrome refers to the commonly occurring disorder comprising central obesity, systemic hypertension, insulin resistance, atherogenic dyslipidemia specifically hypertriglyceridemia and reduced levels of high-density lipoprotein cholesterol [1, 2]. In the general population, MetS increases the risks of cardiovascular disease (CVD), type 2 diabetes mellitus (T2DM), stroke and cardiovascular mortality [3]. The presence of metabolic syndrome components in Type 1 diabetes mellitus (T1DM) patients is frequent and is associated with an increased incidence of chronic complications and mortality [4–6]. Studies suggest that numerous risk factors are responsible for metabolic syndrome in T1DM patients including older age, higher body mass index and glycosylated hemoglobin level [7], elevated diastolic blood pressure and waist circumstance [8] and alcohol consumption [5].

A number of expert groups have developed many different types of clinical criteria for the diagnoses of metabolic syndrome, none of which has gained unanimous acceptance. The first proposal came in 1998 from World Health Organization (WHO) [9], followed by the European Group for Study of Insulin Resistance (EGIR) [10]. In 2001, the National Cholesterol Education Program (NCEP) Adult Treatment Panel III (ATP III) devised a definition for the metabolic syndrome [11], which was updated by the American Heart Association and the National Heart Lung and Blood Institute (modified NCEP) in 2005 [12]. Another set of criteria for the clinical diagnosis of metabolic syndrome has been published in 2005 by the International Diabetes Federation (IDF) [13]. In 2009, a Joint Interim Statement (JIS) was agreed and released by many organizations [14]. Although these organizations have proposed measuring the same components, they have suggested different combinations and different cut-off points. Therefore, the prevalence of MetS varies according to the diagnostic criteria used (Table 1).

The prevalence of the metabolic syndrome ranges from 20 to 25% in the adult population [15, 16] and 0 to 19.2% [17] in children; but it can reach almost 80% in type 2 diabetes patients [18]. Previous studies reported that the prevalence of MetS in T1DM patients varies between 3.2% in Poland [19] and 57.1% in Finland [20] depending on the study population characteristics and the diagnostic criteria used [4]. Even though there are review articles published on the prevalence of MetS among T1DM patients [4, 21], they failed to quantitatively estimate the overall pooled prevalence. Therefore, this study aimed to estimate the pooled prevalence of metabolic syndrome among patients with type 1 diabetes mellitus.

**Table 1:** The definitions of metabolic syndrome

		WHO [9]	NCEP [11]	Modified NCEP [12]	IDF [13]	JIS [14]
Criteria for diagnosis of MetS		Diabetes diagnosis or FBG ≥ 110 mg/dL or IR with ≥ 2 of the following	Presence of any 3 of 5 of the following	Presence of any 3 of 5 of the following	WC: > 94 cm (men); > 80 cm (women) with the presence of ≥ 2 of the following	Presence of any 3 of 5 of the following
Hyperglycemia	Fasting glucose	Already required	≥ 110 mg/dl	≥ 100 mg/dL or on Rx for elevated glucose	≥ 100 mg/dl or diagnosed diabetes	≥ 100 mg/dl or diagnosed diabetes
Dyslipidemia	TG:	> 150 mg/dl	≥ 150 mg/dl	≥ 150 mg/dL or on TG Rx	≥ 150 mg/dl or on TG Rx	≥ 150 mg/dl or on TG Rx
	HDL-C:	M: < 35 mg/dl F: < 40 mg/dl	M: < 40 mg/dl F: < 50 mg/dl or on HDL-C Rx	M: ≤ 40 mg/dL F: ≤ 50 mg/dL or on HDL-C Rx	M: < 40 mg/dl F: < 50 mg/dl or on HDL-C Rx	M: < 40 mg/dl F: < 50 mg/dl in women or on HDL-C Rx
Hypertension	Blood pressure	≥ 140/90 mmHg	≥ 130/85 mmHg	SBP: ≥ 130 mmHg or DBP: ≥ 85 mmHg or on hypertension Rx	SBP: ≥ 130 mmHg or DBP: ≥ 85 mmHg or on hypertension Rx	SBP: ≥ 130 mmHg or DBP: ≥ 85 mmHg or on hypertension Rx
Obesity	WC		M: > 102 cm F: > 88 cm	M: ≥ 102 cm F: ≥ 88 cm	Already required	Ethnic dependent
	Waist/hip ratio:	M: > 0.9 F: > 0.85 or BMI > 30 kg/m^2^				
Other		UAE ≥ 20 μg/min				

BMI: body mass index; DBP: diastolic blood pressure; F: female; FBG: fasting blood glucose; HDL-C: high density lipoprotein cholesterol; IDF: International Diabetes Federation; IR: insulin resistance; JIS: Joint Interim Statement; M: male; NCEP: National Cholesterol Education Program; Rx: treatment; SBP: systolic blood pressure; TG: triglyceride; UAE: urinary albumin excretion; WHO: World Health Organization; WC: waist circumstance

## Methods

### Protocol and registration

The study protocol is registered on PROSPERO with reference number: CRD42020213435. To ensure scientific rigor, the Preferred Reporting Items for Systematic Reviews and Meta-Analysis (PRISMA) guideline was used [22]. The completed checklist is provided as Additional file 1.

### Search strategy

The searches were carried out in Medline via PubMed, CINAHL, ScienceDirect, OVID and other supplementary sources including Google Scholar, ResearchGate and African Journals Online (AJOL). Advanced search strategies were applied in major databases. We used the following key search terms: “metabolic syndrome”, “syndrome X”, “insulin resistance syndrome”, “type 1 diabetes”, “autoimmune diabetes”, “insulin dependent diabetes” and “double diabetes”. The key terms were used in combination using Boolean operators like “OR” or “AND” (see Additional file 2). We also added a hand-search of bibliographies of the included studies for additional references and grey literature. Articles published in subscription based journals were accessed through HINARI. The date of the final search for literatures was October 16, 2020.

### Study selection

All observational studies that reported prevalence of metabolic syndrome among T1DM patients and fulfilled the following criteria were entered into the analysis: (1) original studies; (2) human studies; (3) published between January 1, 2005, and October 16, 2020. Non-English articles were also included by translating using Google translate. Studies were excluded if: (1) not fully accessible; (2) possessed a poor quality score as per the stated criteria; (3) duplicate studies, short communications, case reports, conference abstracts, and letters to editors and/or (4) failed to measure the desired outcome of interest.

The presence of MetS in the individual studies was considered if defined according to one of the following mostly accepted criteria; (1) JIS; (2) IDF; (3) modified NCEP; (4) NCEP and (5) WHO (Table 2). Furthermore, if more than one diagnostic criteria of MetS were used in a study, the first choice was the JIS followed by IDF.

**Table 2:** Characteristics of studies included for systematic review and meta-analysis

Authors	Country	Design	DM duration^a^ (in years)	Age^a^ (in years)	Study participants^b^	Diagnostic criteria	MetS cases	Prevalence (%)			Quality rating (NOS)
								Overall	Male	Female	
Ahola et al. [20]	Finland	CS	NR	45 ± 13.5	791	JIS	452	57.1	80.7	56.2	Moderate
Barros et al. [29]	Brazil	CS	15.5 ± 9.3	30 ± 12	1662	IDF	469	28.2	19.8	35.1	High
Billow et al. [44]	India	CS	NR	15.37 ± 13.5	451	JIS	100	22.2	22.8	21.3	Moderate
Blaslov et al. [27]	Croatia	CS	22.17 ± 11.7	45.08 ± 11.87	77	IDF	26	33.8	36.2	30.0	Moderate
Bonadonna et al. [36]	Italy	CS	16 ± 11	48 ± 17	638	IDF	260	40.8	34.1	47.4	Moderate
Chillarón et al. [7]	Spain	CS	16.7 ± 12.9	39.7 ± 13.2	91	Modified NCEP	29	31.9	32.1	31.6	High
Davis et al. [30]	Australia	Cohort	NR	42.0 ± 15.7	127	IDF	50	39.4	NR	NR	High
Ferreira-Hermosillo et al. [48]	Mexico	CS	17 (11–25)	28 (22–37)	140	JIS	61	44.0	NR	NR	Moderate
Ghosh et al. [41]	Scotland	CS	19.04 ± 12.9	43.78 ± 18.9	365	WHO	112	30.7	56.3	19.4	Moderate
Hawa et al. [49]	Europe countries^c^	CS	18.2 ± 11.7	43.8 ± 9.8	288	NCEP	92	31.9	NR	NR	High
Huo et al. [31]	China	CS	4 (1–8)	16 (9–28)	754	IDF	76	9.0	7.5	12.5	Moderate
Kilpatrick et al. 2007 [32]	USA	Cohort	NR	26.5 ± 7.5	1337	IDF	291	21.7	NR	NR	High
Köken et al. [33]	Turkey	CS	4.6 ± 3.3	13.8 ± 2.8	200	IDF	21	10.5	NR	NR	Moderate
Lee et al. 2020 [50]	Australia	CS	18.5 ± 12.5	44.3 ± 15.6	2120	WHO	643	30.0	31.8	28.5	High
Łuczyński et al. 2011 [19]	Poland	CS	4.4 (2.1–7.0)	13.6 (10.2–15.9)	500	IDF	16	3.2	NR	NR	Moderate
McGill et al. [43]	Australia	CS	NR	NR	427	WHO	64	15.0	NR	NR	High
Merger et al. [28]	Germany/Austria	Cohort	15.66 ± 13.1	38.36 ± 18.7	31,119	NCEP	7926	25.5	25.8	25.0	Moderate
Mollo et al. [51]	Spain	CS	0.8(0.5–3.25)	45.5 ± 11.9	78	NCEP	11	15.5	NR	NR	Moderate
Nádas et al. [34]	Hungary	CS	18.0 ± 11.1	35.6 ± 11.6	533	IDF	193	36.2	32.8	39.4	High
Pambianco et al. [35]	USA	Cohort	NR	NR	514	IDF	43	8.0	2.7	12.7	High
Rodrigues et al.[52]	Brazil	CS	16.5 ± 9	34.8 ± 11	261	NCEP	35	13.4	10.9	16.1	Moderate
Saki [37]	Iran	CS	4.4 ± 2.8	12.38 ± 4.2	87	IDF	26	29.9	25.6	22.9	Moderate
Santos et al. [38]	Brazil	CS	16.8 ± 10	32.5 ± 10	101	IDF	32	32.0	NR	NR	Moderate
Soliman et al. [8]	Egypt	CS	5.74 ± 3	13.38 ± 2.17	160	IDF	21	13.1	7.8	18.1	Moderate
Szadkowska et al. [39]	Poland	CS	6.2 ± 4.2	14.8 ± 2.4	163	IDF	12	7.4	6.6	8.3	Moderate
Thorn et al. [53]	Finland	CS	21.9 ± 1.8	37.2 ± 1.3	2415	NCEP	944	39.1	38.2	40.1	Moderate
Valerio et al. [40]	Italy	CS	8.4 ± 3.9	17.3 ± 0.9	412	IDF	39	9.5	3.7	16.1	High

N: number of T1DM patients participated in the study; MetS: metabolic syndrome; NOS: Newcastle Ottawa Scale; T1DM: Type 1 diabetes mellitus; DM: diabetes mellitus; CS: cross-sectional; NECP: National Cholesterol Education Program Expert Panel; IDF: International Diabetes Federation; WHO: World Health Organization; JIS: Joint Interim Statement; NR: Not Reported; NA: not applicable;

^a^Data are in mean (± standard deviation) or median (inter quartile range); ^b^ only T1DM patients; ^c^Ireland, France, Spain, Italy and England;

Articles that fulfilled inclusion criteria were imported into Endnote9 citation manager. After deleting duplicate records between different bibliographic databases, the remaining titles and abstracts were independently reviewed by two authors (RB and ZA) to identify potentially eligible articles that required a full appraisal. In cases of multiple publications from the same study or overlapping data, preference was given to the most recent one or the one with the most inclusive information. Consensus was achieved for any discrepancies in study eligibility selection through discussion with other authors (AA and or MS).

### Data extraction and quality assessment

Data from the selected studies were recorded into the pre-prepared MS Excel extraction form (see Additional file 3). For each included study, the following data were extracted: first author, publication year, country, mean age, mean diabetes duration, study design, study participants, MetS diagnostic criteria, and outcome of interest (MetS cases and prevalence of MetS). Data not presented in the articles were accessed by contacting the corresponding author or, if possible, were calculated from the available data. The methodological quality of each included study was assessed using the modified Newcastle–Ottawa scale (see Additional file 4) [23]. Each article’s quality was graded as ‘high’ if score 8–10; ‘moderate’ if score 5–7; and ‘low’ if score < 5 points. Studies were included in the analysis if they scored ≥ 5 out of 10 points.

Furthermore, data extraction and quality checks were independently performed by two authors (RB and AA). Any disagreements were resolved by discussion and if that fails, other authors (ZA and MS) called on to adjudicate the final judgments.

### Statistical analysis

Meta-analysis using DerSimonian and Laird random-effects model was utilized to obtain the pooled prevalence of metabolic syndrome among T1DM due to expected heterogeneity among studies. The pooled effect size (i.e. prevalence) with a 95% confidence interval (CI) was generated and presented using a forest plot. Heterogeneity between studies was assessed using the Cochran's Q and I^2^ statistic. I^2^ values of 25%, 50%, and 75% were considered to represent low, moderate and high heterogeneity, respectively [24]. Potential sources of heterogeneity were investigated by subgroup and meta-regression analysis. In addition, potential outliers were investigated in a sensitivity analysis by omitting each study at a time. We also used Funnel plot symmetry, Egger’s regression test and Begg’s test for evaluating the possibility of publication bias [25, 26]. P-value < 0.05 was considered statistically significant. All statistical analyses were performed using Stata/MP 16.0 (StataCorp, College Station, TX, USA).

## Results

### Search results

**Fig. 1:**
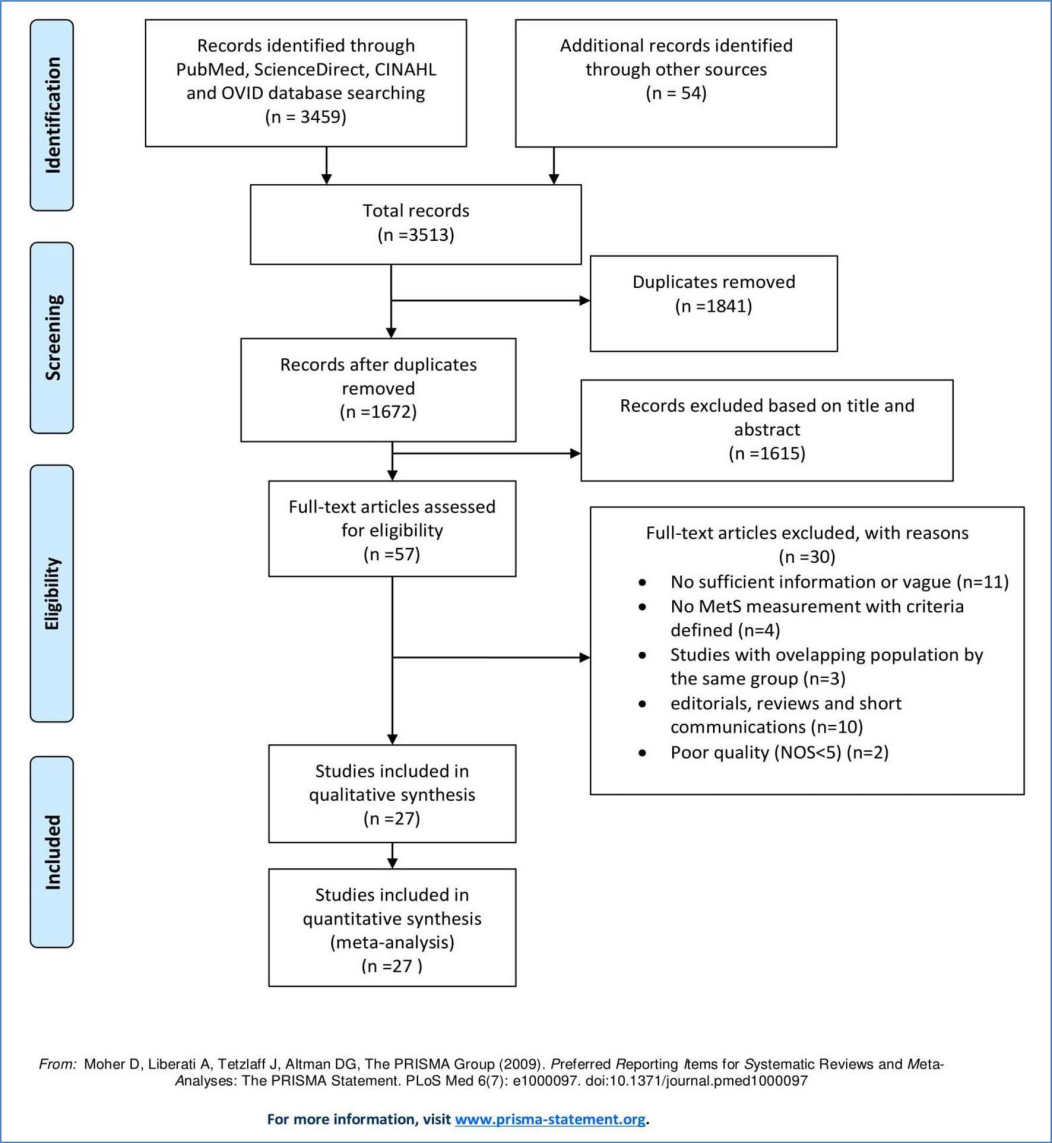
Flow diagram of study selection

Our comprehensive search strategy owns us a total of 3459 articles. Of these, 445 from Medline via PubMed, 1637 from ScienceDirect, 669 from CINAHL, 737 were from Ovid interface and 54 were found through a manual search. After excluding duplicate publications, 1672 articles remained. About 1615 articles were excluded after reading the titles and abstracts based on the pre-defined eligibility criteria. Out of them 57 articles were screened for further assessment. Finally, 27 articles were included in the synthesis and analysis (Fig. 1).

### Baseline characteristics of the included studies

From the studies included in the final analysis, 23 (85.2%) of them were cross-sectional and 7 (14.8%) were prospective cohort studies. The sample size of the included studies ranged from 77 [27] to 31,119 [28] with a total number of 45,811 participants. Twenty three countries were represented in this review. Most of the studies were reported from Europe 13 (48.1%) followed by Asia 4 (14.8%). The rest were reported from South America 3 (11.1%), North America 3 (11.1%), Australia 3 (11.1%) and Africa 1 (3.7%). In terms of diagnostic criteria, a total of 15 studies [8, 19, 27, 29–40] used IDF, 5 studies [24, 37–40] used NCEP, 3 studies [41–43] used WHO, 3 studies [20, 44, 45] used JIS and 1 study [7] used modified NCEP (Table 2).

### Prevalence of metabolic syndrome

**Fig. 2:**
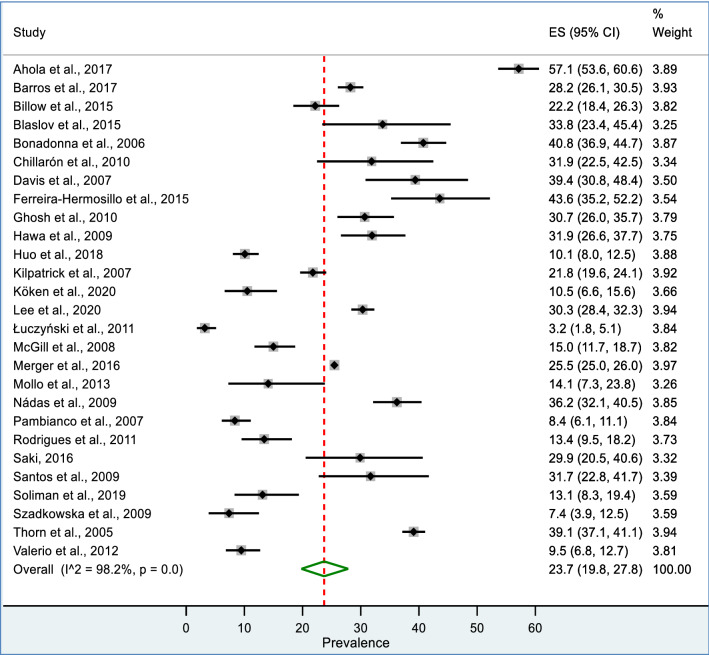
Forest plot of showing pooled prevalence of MetS among patents with T1DM

The overall pooled prevalence of MetS among patients with T1DM was 23.7% (95% CI: 19.8, 27.8) with substantial heterogeneity (I^2^ = 98.2%; P value of < 0.001). Individual study prevalence estimates ranged from 3.2 to 57.1% whereas studies individual weight was from 3.25 to 3.97%. Figure 2 presents the Forest Plot derived from the meta-analysis.

### Prevalence of metabolic syndrome by gender

**Fig. 3:**
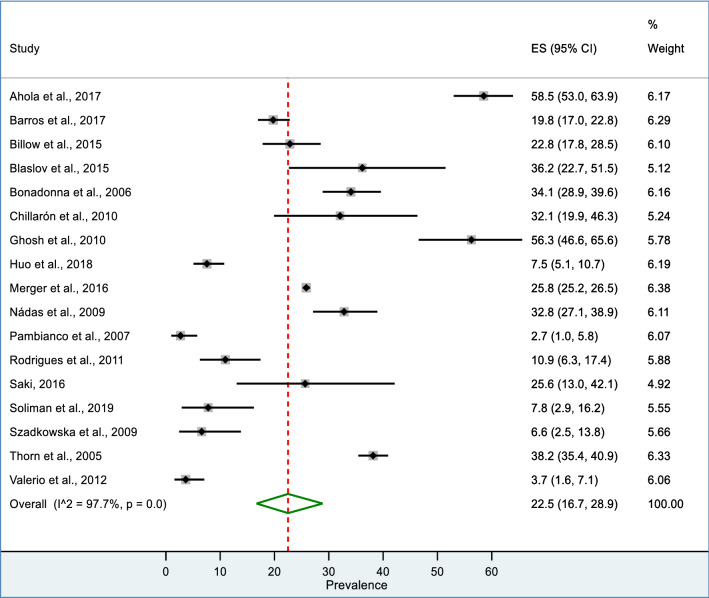
Forest plot of showing pooled prevalence of MetS among male T1DM patents

**Fig. 4:**
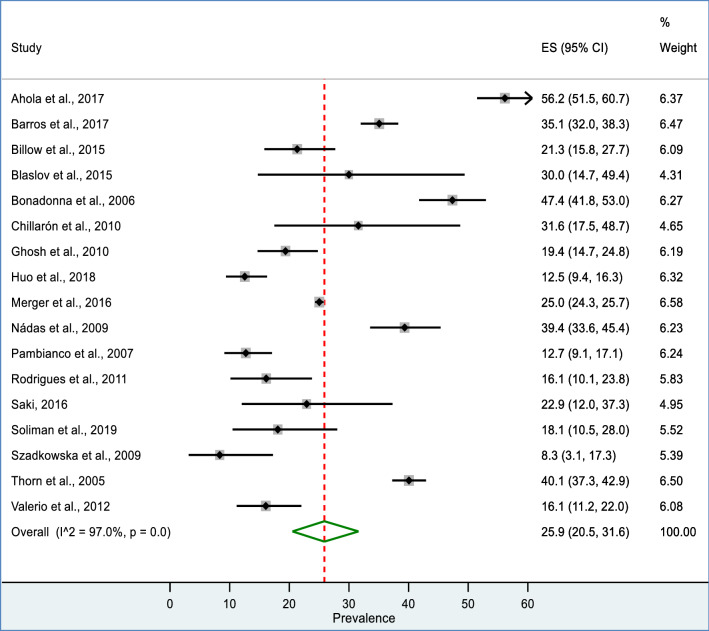
Forest plot of showing pooled prevalence of MetS among female T1DM patents

Seventeen studies (N = 40,493) had separate data on the prevalence of MetS for males and females. The pooled prevalence for males was 22.5% (95%: CI 16.7 to 28.9%) (Fig. 3) while, it was 25.9% (95% CI: 20.5 to 31.6%) for females (Fig. 4). A significant heterogeneity was found in both males (I^2^ = 97.7%; P < 0.001) and females (I^2^ = 97.0%; P < 0.001).

### Subgroup and sensitivity analysis

To identify the source of heterogeneity across the included studies, subgroup analyses were done for publication year, study design, geographical region, diagnostic criteria, and sample size. Time based subgroup revealed that the prevalence of MetS from 2015 to October 2020 (26.6%) was higher when compared with 2005 to 2014 (21.8%) whereas the results of subgroup analysis based on geographical region showed the highest prevalence was from Australia (27.3%) and the least was from Africa (13.1%). Another subgroup analysis with diagnostic criteria showed the highest prevalence, 40.5% (95% CI 17.7, 65.6), of MetS was observed with JIS whereas the lowest, 19.8% (95% CI 13.6, 26.8), was observed with IDF. Furthermore, the pooled prevalence of MetS in studies conducted by cross sectional and cohort study design was 24.0% (95% CI 18.3, 30.1) and 22.2% (95% CI 14.9, 30.4) respectively. Results of the subgroup analysis are depicted in Table 3.

**Table 3:** Subgroup meta-analysis of metabolic syndrome prevalence among Type 1 diabetes mellitus patients

Subgroup	No. of studies	Sample size	Prevalence (95% CI)	Heterogeneity			
				Q value	df	P value	I^2^
Publication year							
2005–2014	16	8250	21.8 (15.1,29.4)	870.3	15	< 0.001	98.3%
2015–2020	11	37,561	26.6 (20.8, 32.7)	566.5	10	< 0.001	98.2%
Study design							
Cross-sectional	23	12,714	24.0 (18.3, 30.1)	1258.6	22	–	98.3%
Cohort	4	33,097	22.2 (14.9, 30.4)	131.3	3	–	97.7%
Geographical region							
Europe	13	37,470	26.2 (19.3,33.7)	994.8	12	< 0.001	98.8%
North America	3	1991	22.6 ( 9.5, 39.4)	–	2	–	–
South America	3	2024	23.7 (13.6,35.6)	–	2	–	–
Asia	4	1492	17.0 (9.5, 26.1)	46.9	3	< 0.001	93.6%
Australia	3	2674	27.3 (15.7, 40.7)	–	2	–	–
Africa	1	160	13.1 (8.3, 19.4)	–	0	–	–
Sample size							
≤ 300	12	1773	23.8 (16.6, 31.8)	154.0	11	< 0.001	92.9%
> 300	15	44,038	23.7 (18.7, 29.2)	1271.4	14	< 0.001	98.9%
Diagnostic criteria							
WHO	3	2912	25.0 (15.9, 35.4)		2	–	–
NCEP	5	34,161	24.8 (17.3, 33.1)	231.1	4	< 0.001	98.3%
Modified NCEP	1	91	31.9 (22.5, 42.5)	-	0	–	–
IDF	15	7265	19.8 (13.6, 26.8)	666.0	14	< 0.001	97.9%
JIS	3	1382	40.5 (17.7, 65.6)		2	–	–

**Fig. 5:**
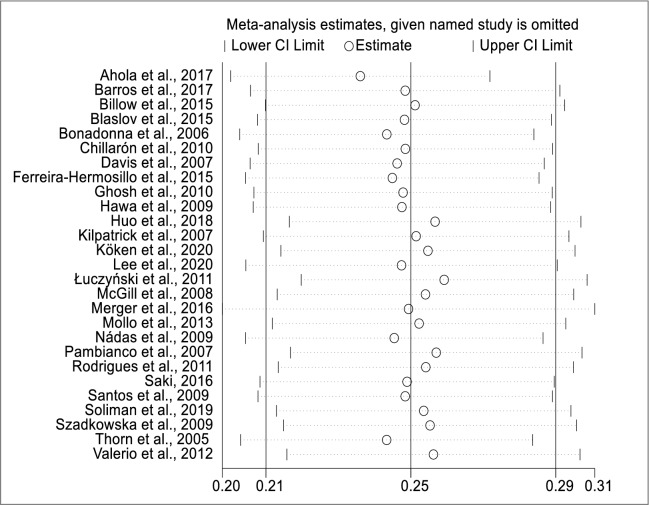
Results of sensitivity analysis

**Fig. 6:**
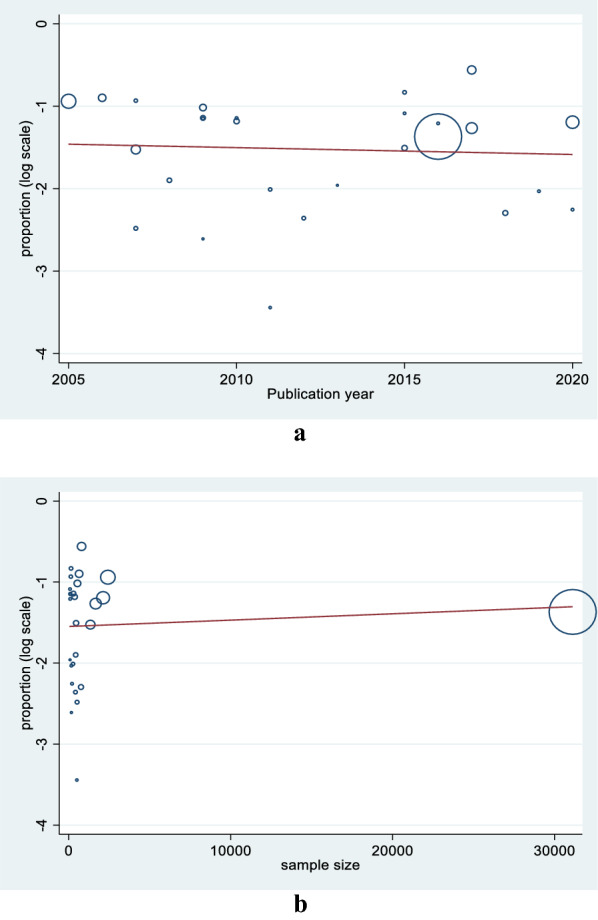
Univariate meta-regression model using **a** publication year **b** sample size

To identify a single study influence on the overall meta-analysis, sensitivity analysis using the leave-one-out approach was performed and the result showed that there was no strong evidence for the effect of a single study on the overall meta-analysis result (Fig. 5). To further explore the heterogeneity observed in the study, we carried out meta-regression. Univariate meta-regression revealed that publication year (regression coefficient = 0.99; P-value = 0.77) and sample size (regression coefficient = 1.00; P-value = 0.71) are not a source of heterogeneity (Fig. 6).

### Publication bias

**Fig. 7:**
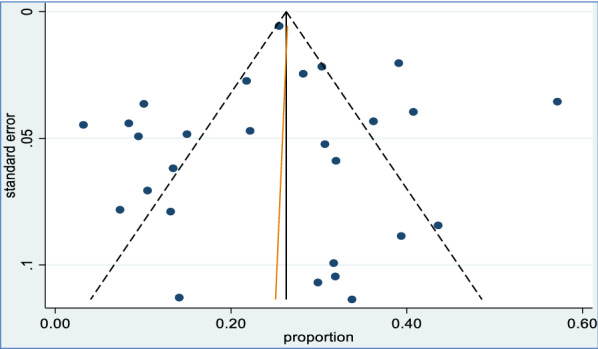
Publication bias using funnel plot

The funnel plot (Fig. 7) was symmetric and Egger’s regression test (P = 0.87) as well as Begg’s test (P = 0.90) provided no evidence of publication bias.

## Discussion

To the best of our knowledge, this study is the first of its kind to quantitatively pool the prevalence of MetS among T1DM. Meta-analysis of 27 original studies with 45,811 study participants showed that approximately 23.7% of patients with T1DM had MetS. As per meta-analysis of 17 studies, the pooled prevalence of MetS in female T1DM patients (25.9%) was slightly higher than male T1DM patients (22.5%). High degrees of variability of prevalence of MetS among patients with T1DM were reported in studies included in this meta-analysis. The highest prevalence of MetS was reported in Finland (57.1%) (20) whereas the lowest prevalence was reported in Poland (3.2%) [19]. This variation might be due to differences in diagnostic criteria used [46], study design, sample size and characteristics of the population participated in the studies.

The results of subgroup analysis based on geographical region showed that the highest prevalence was from Australia (27.3%) and the least was from Africa (13.1%). The possible explanations for this variation might be due to socioeconomic and sociocultural differences between the populations. Another possible explanations for this variation might be differences in the diagnosis definition used, incomparable number of studies from regions and variations in the prevalence of MetS in the general population of the respective regions.

Time- based subgroup revealed that the prevalence of MetS from 2015 to October 2020 (26.6%) was higher when compared with 2005 to 2014 (21.8%). This could indicate the increasing trend of MetS among type 1 DM patients worldwide. This increased prevalence probably due to the rising prevalence of MetS as a result of the obesity epidemic in the general population [47]. Consistent with our result, a study conducted in United Kingdom indicated a significant increasing trend of MetS among T1DM patients [32].

Of the five definitions used by studies included in this review, the estimated prevalence was highest based on JIS (40.5%) and lowest based on IDF consensus (19.8%). This high discrepancy may be due to abdominal obesity criteria which is not mandatory in JIS definition. Similar findings in variation of MetS prevalence per diagnostic criteria were also reported in many studies conducted in different corners of the world [30, 33–36, 38, 46]. To solve this problem, an internationally accepted practical and uniform definition of MetS has to be established.

This systematic review and meta-analysis indicates that diabetic patients are facing an epidemic of MetS, and thus, clinicians should pay more attention to the cardiometabolic profiles of diabetic patients and develop targeted strategies against components and risk factors of MetS. We hope that the findings of the current review provide valuable information to the policymakers, National Health Bureaus and other concerned bodies about global and regional prevalence of MetS among T1DM patients. These also can be used for future complementary researches.

## Limitations of the study

This study has a few potential important limitations. First of all, different types of definitions used to diagnose MetS in the included studies may affect the calculation of the pooled prevalence. Additionally, studies from developing countries are rare, which will impact the estimation of the average prevalence of MetS globally. Furthermore, there is substantial heterogeneity observed between studies that may affect the interpretation of the results. Sources of heterogeneity might be from age category and diabetes duration as well as insulin dose which were not investigated due to the incomplete data contained in original articles.

### Conclusion

Nearly a quarter of the T1DM patients were affected by MetS. Therefore, more attention should be paid to the prevention and control of MetS to ameliorate a further increase in the epidemic and for the reduction of the morbidity and mortality associated with MetS among T1DM patients.

## Supplementary Information

**Additional file 1. ** Completed PRISMA checklist.

**Additional file 2. ** Search strategy.

**Additional file 3. **Extracted data

**Additional file 4. **Quality assessment score.

## Data Availability

The datasets supporting the conclusions of this article are included within the article and its additional files.

## Abbreviations

DM: Diabetes mellitus
IDF: International Diabetes Federation
MetS: Metabolic syndrome
T1DM: Type 1 diabetes mellitus
NCEP: National Cholesterol Education Program
NOS: Newcastle Ottawa Scale
WHO: World Health Organization
JIS: Joint Interim Statement
