# Supplementary material for: Global prevalence of metabolic syndrome among patients with type I diabetes mellitus: a systematic review and meta-analysis
Source: Diabetol Metab Syndr. 2021 Mar 2;13:25. doi: 10.1186/s13098-021-00641-8 (PMC7923483; doi:10.1186/s13098-021-00641-8)
Supplement: Supplementary file 2 — Additional file 2. Search strategy. [file 13098_2021_641_MOESM2_ESM.docx]

Search terms used for final search of databases, 16 October 2020

| Searches | #1 | #2 | #3 |
| --- | --- | --- | --- |
| Search terms | "Metabolic syndrome" OR "insulin resistance syndrome" OR "syndrome X" | "Type 1 Diabetes Mellitus" OR "autoimmune diabetes" OR "insulin-dependent diabetes mellitus" | #1 AND #2 |
| Science Direct | 38,672 | 10,435 | 1608 |
| CINAHL | 17,869 | 14,333 | 669 |
| PubMed | 42,030 | 37,023 | 445 |
| Ovid | 1251 | 699 | 737 |
